# Impact of Remote Patient Monitoring Platform on Patients With Moderate to Severe Persistent Asthma: Observational Study

**DOI:** 10.2196/51065

**Published:** 2023-12-28

**Authors:** Denzil Reid, Jyotsna Mehta, Karim Anis, Shail Mehta

**Affiliations:** 1 Baystate Health Springfield, MA United States; 2 Keva Health Lexington, MA United States

**Keywords:** asthma, remote patient monitoring, virtual care solution, respiratory care, chronic disease management, spirometry, telehealth, management, chronic disease, virtual care, patient monitoring, respiratory, respiratory care, respiratory illness, patient monitoring tool, tool, decision-making

## Abstract

**Background:**

Asthma is one of the most common respiratory diseases, with an ever-growing health care burden. Remote patient monitoring (RPM) has gained increasing importance in the respiratory care area with the outbreak of the COVID-19 pandemic. In this pilot study, we introduced a novel platform that remotely monitors patients with chronic respiratory illnesses using Centers for Disease Control and Prevention guidelines to reduce hospitalizations and emergency department visits.

**Objective:**

This study aimed to understand patient and physician engagement with a new virtual care solution (KevaTalk app and Keva365 platform) and the value, for both patients and providers, of using an RPM tool. We assessed real-world use of the platform from both physician and patient perspectives and the impact of devices on engagement and monitoring.

**Methods:**

Participants with a history of moderate to severe persistent asthma, seen by a pulmonologist at a hospital, were included in this study. The inclusion criteria involved being aged ≥18 years and having access to an Android or iOS mobile device with internet. We provided patient questionnaires to assess the app’s usefulness and evaluate its features. We monitored remote spirometry and oximetry data, app check-ins, alerts, and escalations during this study’s time window. Data were reviewed daily and predetermined criteria were set to escalate for physician review based on the patient’s symptoms and objective data.

**Results:**

Overall, 25 patients were included in this pilot. The mean age was 57 (SD 10.7) years and a majority (n=23, 92%) were female. A baseline questionnaire, which was used to rate the app, indicated that the ease of check-in and ease of modification to the patient’s asthma plan were the 2 highest rated features. In total, 2066 check-ins (1550 green, 506 yellow, and 10 red check-ins) and 1155 spirometry sessions were recorded during this 3-month period. Further, 64% (14/22) and 91% (20/22) of patients were found to have peak flows in their red and yellow zones at least once, respectively. During the course of this study, 484 alerts were recorded and evaluated by the team, of which 37.2% (n=180) required an escalation to the physician; this included a transfer to a medical facility, change in respiratory medication, or further education.

**Conclusions:**

In this pilot study, we demonstrated the feasibility of implementing a novel RPM platform in patients with asthma. Our platform showed high patient engagement and satisfaction and provided physicians with real-time subjective data to evaluate patients remotely that aids in clinical decision-making. The escalations prevented patients from having an exacerbation or flare up, which led to the prevention of an emergency department visit. Continuous monitoring of chronic disease has benefits over episodic monitoring. It allows for improved quality of life, better outcomes, and huge health care savings.

## Introduction

Asthma is one of the most common respiratory diseases affecting both children and adults. It is estimated to affect 300 million people [1], and by 2025, it is expected that an additional 100 million individuals will have asthma [2]. Moreover, asthma kills roughly 1 in every 250 people on the planet. In total, 1 in 13 individuals in the United States have asthma; according to the Centers for Disease Control and Prevention (CDC), 4,226,659 children and 21,030,479 adults are affected by asthma, which makes up 7.8% of the population. There is also a clear gender disparity for this condition: 9.7% of women and 6.2% of men have asthma [3]. Moreover, the prevalence of asthma attacks among people with current asthma is 39.4% [4], causing 1,835,901 hospital emergency department visits and 169,330 hospital inpatient stays. The mean mortality rate (deaths per million people) published by the CDC is given to be 10.6 (SE 0.18), which is 2.0 (SE 0.16) in children and 13.1 (SE 0.22) in adults; the rate is the highest among the 65+ years of age population (27.1, SE 0.70); and the mortality rate is directly proportional to age. While there are numerous programs that aim to help patients with asthma, such as the CDC’s National Center for Environmental Health, which plans, directs, and coordinates a program to protect the American people from environmental hazards; the National Environmental Public Health Tracking Network, which is a system of integrated health, exposure, and hazard information and data from a variety of national, state, and city sources; and the CDC’s National Institute for Occupational Safety and Health, which provides information on work-related asthma, there is more that can be done to help patients manage their condition better, especially remotely.

An essential part of remote health care is the use of technology to monitor patients outside of hospital settings. There are many advantages of internet of things–based health monitoring systems, such as continuous patient monitoring and real-time illness detection; reducing the need for and cost of hospitalization, use of technologies, and emergency medical assistance; and obtaining more accurate data [5].

Remote patient monitoring (RPM) is a method of collecting health-related data from patients who are in a remote location and electronically transmitting it to health care providers for evaluation and consultation. RPM or telemonitoring aims at improving patient care through digitally transmitted health-related data. This allows early detection of disease decompensation and intervention, facilitates patient education, and improves the patient**-**physician relationship [6]. Continuous care is intended to help the patient stay at their residential facility through a health crisis, rather than being moved to a hospital.

Our platform (Keva365 Remote Respiratory Care platform; Keva Health Inc) remotely monitors patients with chronic respiratory illnesses using CDC guidelines to reduce hospitalizations and emergency department visits. It does this by encouraging patients to follow physician-provided action plans and provides just-in-time education when they are at home. The platform provides automated virtual check-ins where patients can log their symptoms. Devices such as handheld spirometers (MIR Spirobank Smart) and oximeters were provided to the patients; these devices were then connected to a mobile app through Bluetooth to monitor and track the patient’s day-to-day data. The goal of the pilot was to understand patient and physician engagement with our platform, as well as understand the value, for both patients and providers, of using an RPM tool. Moreover, there seems to be a lack of published data on digital interventions for the management of asthma, which is why the goal for this pilot was to contribute to this underrepresented space.

## Methods

### Study Design

Participants included in this study were those with moderate to severe persistent asthma. They were seen by a pulmonologist at Baystate Noble Hospital within the division of pulmonary care and critical medicine located in Springfield, Massachusetts. Patients were offered the Keva program and recruited during their specialty care visit with their pulmonologist. The participants had to be 18 years of age or older, had access to an Android or iOS mobile device with internet connectivity, and had an active email address. Further, the patients were required to have no visual, cognitive, or other impairments that may prevent the patient from being able to participate. Patients were also required to have English-language proﬁciency and literacy (sixth-grade reading level). Patients were excluded from the pilot for any conditions that would prevent them from using an app during the duration of the pilot (provider discretion).

A baseline questionnaire was provided to the patients at the start of this study (Section S1 in Multimedia Appendix 1). A tobacco use questionnaire was also provided to the patients at enrollment (Section S2 in Multimedia Appendix 1). Patients were asked to fill the Asthma Therapy Attack Questionnaire (ATAQ) once a month through the app (Section S3 in Multimedia Appendix 1). The ATAQ is a 4-question scale that assesses the limitation of activity, nocturnal awakening, self-assessment of asthma control level, and rescue inhaler use [7].

Remote spirometry and oximetry were provided to this study’s participants. These spirometers were able to capture several different parameters of lung function such as forced vital capacity, peak expiratory flow, forced expiratory volume in 1 second, forced expiratory volume in 6 seconds, and forced expiratory volume in 1 second or forced vital capacity.

Registered respiratory therapists set up 45-minute onboarding video calls that were arranged for each participant after enrollment. During these video calls, participants were taught how to use the spirometer and oximeter. Further, the respiratory therapist ensured the participant was able to check their asthma action plan and enter and remove their medications in the app.

**Figure 1:**
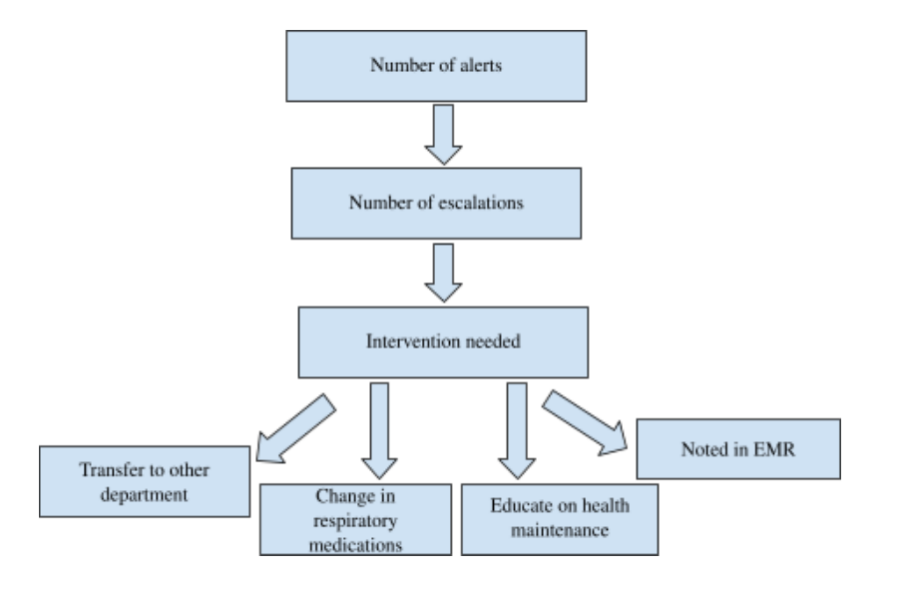
Flowchart for alerts and escalations from remote monitoring of patients with asthma in the US observational study. EMR: electronic medical record.

Each week, a report was generated, which included engagement statistics, use of devices, percentage of spirometer session, and percentage of questionnaires filled up by physicians during that week. Remote monitoring protocols were set up for patients, which included specific requirements for escalations and alerts with pulmonologists (Figure 1). These protocols included instructions on alerts generated through the use of the app and reported to the office. The care team monitored the number of check-ins and data generated from remote spirometry. Engagement was assessed by averaging weekly check-ins, alerts, and number of spirometry sessions by the patients. In total, 2 yellow check-ins or 1 red check-in prompted a call to the patient first. The care team made notes and then informed the doctor’s office for further follow-up. The office provided follow-up on the escalations generated by the Keva nurse in order to ensure the patients were given the needed interventions.

During this study, a total of 9 interviews were conducted by a health economics and outcomes research consulting agency. Patients were selected after they had completed 3 months of the program. An excerpt summary included the users’ use of this app, spirometer data, and referral to friends. An interview guide was created and provided to the patients, and Zoom (Zoom Video Communications) interviews were conducted. Each interview lasted around 1 hour and patients were compensated for their time.

The research team consisted of the pulmonologist under whom the patients were seen, nurses who helped manage alerts, respiratory therapist who conducted the onboarding video calls, and a medical assistant who entered the alerts in the emergency department and followed up with the patient as needed.

### Ethical Considerations

All study procedures were approved by the Baystate Techspring review team at Baystate Health (approval 2021-R-015). This study was approved by the compliance and IT team at Baystate Health. Each study participant read and signed an informed consent form outlining the purpose, risks, and benefits of this study; participants had the opportunity to ask questions and seek clarification from this study’s staff when reading and signing the form. The informed consent form also described how participation and the resulting data would be kept private and confidential (ie, all study data were deidentified and stored in either locked drawers or on password-protected servers accessible only to this study’s team). Patients received a US $15 gift card for participating in the interview.

## Results

### Patient Demographics

A total of 25 participants were enrolled in this pilot study. The average age of participants was 57 (SD 10.7) years. The majority of our participants were female (23/25, 92%) and of White ethnicity (18/25, 72%; Table 1). A total of 19 patients filled out the questionnaires. The average time filling the questionnaires was 10.7 days. In total, 4 (16%) of the 25 participants were former smokers, while 10 (40%) of them were nonsmokers.

**Table 1:** Patient demographics among patients with asthma in the US observational study (N=25).

Characteristic			Value
Age (y), mean (SD)			57 (10.7)
**Sex, n (%)**			
	Male	2 (8)	
	Female	23 (92)	
**Race and ethnicity, n (%)**			
	Black	2 (8)	
	White	18 (72)	
	Other	5 (20)	
**Smokers, n (%)**			
	Former smoker	4 (16)	
	Never smoked	10 (40)	
	Smoke everyday	1 (4)	
	Did not fill	10 (40)	

### Baseline Questionnaire Results

The patients were asked to fill up a baseline questionnaire in the first 2 weeks after joining this study and were asked to rate each question on a scale of 1 to 5.

In total, 14 patients filled out the baseline questionnaire. On a scale of 1 to 5, patients rated their ease of entering the medication in the app as mean 3.4 (SD 1.6). The average rating for “how easy is it to enter and modify asthma action plans” was 4.6 (SD 0.8). Nearly all of the participants agree that entering daily check-ins was fairly easy (mean 4.7, SD 0.9). Participants also rated how easy it was for them to send a message to the support team from the app; the mean score was 4.5 (SD 1.1). The average rating for learning the app’s functions was 4.2 (SD 1.4) out of 5 (Table 2). In total, 71% (10/14) of the participants had at least 1 visit (office, emergency department, telehealth, or hospital) for asthma in the past 3 months. Further, 21% (3/14) of the participants had 3 or more visits.

**Table 2:** Results of baseline questionnaires among patients with asthma in the US observational study, including app- and disease-specific questions (n=14).

Question type		Value
**App-related questions^a^, mean (SD)**		
	Q1: Ease of entering medications	3.4 (1.6)
	Q2: Ease of modification (asthma action plan)	4.6 (0.8)
	Q3: Ease to check-in	4.7 (0.9)
	Q4: Access to customer support	4.5 (1.1)
	Q5: Resources	4.2 (1.4)
	Q9-Q12: Health care use (average number of asthma-related visits in the past 3 months)	1.5 (1.9)
**Disease-related questions, n**		
	Q6: Understanding of engagement report (number of yes responses)	10
	Q8: Frequency of check-ins	11

^a^These questions were rated on a scale of 1-5, with 1 being not easy to use and 5 being extremely easy to use.

### ATAQ Results

In total, 19 out of 25 participants responded to the ATAQ, with a response rate of 76% (Table 3). Furthermore, 26% (5/19) of the participants miss their work, school, or normal daily activities because of asthma. Only 21% (4/19) of the participants woke up at night because of asthma. Further, 68% (13/19) of the participants believed that their asthma was well controlled in the past 4 weeks. The average highest number of puffs taken by the patients in 1 day is 2 puffs.

**Table 3:** Results of the ATAQ^a^ among patients with asthma in the US observational study (n=19).

Question	Yes responses, n (%)
Q1: Missed daily activities	5 (26)
Q2: Wake up at night	4 (21)
Q3: Asthma under control for the past 4 weeks	13 (68)
Q4: Use inhaler for quick relief	3 (16)

^a^ATAQ: Asthma Therapy Assessment Questionnaire.

### Engagement Results

Out of the 25 patients in our pilot study, the average number of green check-ins for a week was 74.3, the mean number of yellow check-ins for a week was 23, and the average value of red check-ins for a week was 1. There were 636 green check-ins that resulted in further actions by patients, 433 unique spirometry sessions, and 179 trigger alerts navigating patients to content for education and self-management (Table 4)*.*

**Table 4:** Measures for patient engagement using Keva talk app among patients with asthma in the US observational study.

Variable			Value
Total number of patients, n			25
**Green check-ins**			
	Total, n	1550	
	Mean (per week)	74.3	
**Yellow check-ins**			
	Total, n	506	
	Mean (per week)	23	
**Red check-ins**			
	Total, n	10	
	Mean (per week)	1	
Spirometry sessions, n			1155

We monitored check-ins, alerts, and escalations during this study’s time window. Of the 484 alerts generated by the monitoring team, 37.2% (n=180) required escalation. Overall, 1 patient was transferred to the emergency department, medication was changed for 1 patient, and 2 patients required further health maintenance education (Table 5). Spirometry data for the patients are shown in Table 6.

**Table 5:** Alerts and escalations data from remote monitoring among patients with asthma in the US observational study.

Variable	Value
Alerts, n	484
Escalations^a^, n/N (%)	180/484 (37.2)
Noted in emergency department^a^, n	33
Transfer to another department, n/N (%)	1/33 (3)
Health maintenance education, n/N (%)	2/33 (6)
Change in respiratory medications, n/N (%)	1/33 (3)

^a^Patients had repeat escalations, hence n>25.

**Table 6:** Spirometry data from remote monitoring of patients with asthma in the US observational study.

Variable	Value
Patients who performed spirometry, n	22
Spirometry sessions completed, n	1155
Spirometry sessions per patient, mean	53
Patients with peak flow values in the red zone, n/N (%)	14/22 (64)
Patients with peak flow values in the yellow zone, n/N (%)	20/22 (91)
Patients with peak flow values in the green zone, n/N (%)	22/22 (100)

### Interview Results

Multiple patients liked using the Keva app to keep track of their illness and found it easy to connect their spirometer devices. A total of 9 patient interviews were conducted during this study. One of the outstanding things from the interview was that most of the patients liked the daily check-in from the app and entering medications. Patients tended to be engaged when they are feeling well. Patients were very receptive to using the app and device and felt that it was an easy way to track their asthma. Most patients were satisfied with the app since it is easy to use and keeps track of their states of illness. They also felt that it was convenient to use, especially in the COVID-19 pandemic, because they do not need to meet physicians in person and can receive daily updates. The treating physician was able to see the RPM report to allow them to optimize care for the patient and receive alerts when the patient’s condition worsens.

## Discussion

### Principal Findings

In this pilot study, we describe our experience using a novel platform that enables RPM using remote spirometry and oximetry. We demonstrate that an easy-to-use remote monitoring platform in patients with asthma can lead to high patient satisfaction and engagement from the patients. We also demonstrate that such programs can yield meaningful clinical data that can aid physicians in their clinical decision-making.

We found that our platform was rated highly by participants on ease of entering medications, ease of entering their asthma action plan, ease to log symptoms, and technical support. We believe that this leads to a high compliance with the daily tasks of the platform. We had a total of 2066 symptom check-ins during this study period. The mean daily check-in per participant was 83; study compliance was 92% (23/25), defined at the total number of patients checking in regularly using the KevaTalk app. During this 9-month study, 22 (88%) out of 25 participants performed spirometry for an average of 53 daily spirometry sessions per participant. Green, yellow, and red zones were predetermined for each patient based on their baseline peak expiratory flows. All participants logged peak flows in the green zone during this study. Further, 20 patients had peak flows in the yellow zone and 14 had a peak flow measurement in the red zone during this study. Furthermore, 3 consecutive red alerts in the platform (lung function levels lower than usual, symptomatic check-ins, plus looking up their action plan) prompted escalation to the physician’s office for review. Based on these criteria, out of the 484 generated alerts, 37 were escalated to the physician for review. Despite the small number of participants, our platform was able to identify multiple participants who required an intervention. Further, 1 (4%) of the 25 patient was transferred to the emergency department. Maintenance inhaler therapy was changed for 1 patient. In total, 2 patients required further education. The remaining escalations did not require active interventions.

If we were to use the escalations to the office as a proxy for emergency department visits, we would have prevented 37 emergency department visits for the 25 patients in our cohort. Thus, if we extrapolate our findings to the larger asthmatic population, we have the potential to reduce thousands of emergency department visits and hence reduce the economic burden of respiratory diseases on the US health care system. Moreover, this intervention is both cost and time effective and does not require substantial time for the physicians and nurses. Each month, the patient’s RPM report is accessible on the emergency department for the physician to review when the patient makes a visit. The data are also available in real time so any physician or nurse or care team member can access it from the Keva365 platform. The patient can pull up their RPM report when visiting their doctor.

More research needs to be conducted to determine the effect of connected devices and RPM on clinical outcomes in asthma and other respiratory diseases. There is a need for an RPM platform for monitoring patients in between visits to prevent emergency department visits, since oftentimes patients ignore their symptoms until they have exacerbated [8]. Therefore, we aim to expand enrollment in the program to more patients with asthma and other respiratory diseases.

Limitations of this study include technology and access barriers among patients, the fact that large-scale implementation can be challenging in terms of budget (cost of technology and infrastructure to monitor), the need to train providers in the collection and interpretation of results and to keep track of patients’ complaints, as well as the incorporation of the remote patient data into routine clinical practice and workflows.

However, with proper operationalization of the program and collaboration with the physician, these limitations can be overcome, as the value and benefits gained is tremendous for these programs in the short and long term. In conclusion, home self-monitoring of asthma with a connected mobile spirometer is feasible, and it is associated with high patient satisfaction. Active contact between the physicians and appropriately instructed and motivated patients is important for obtaining reliable home spirometry measurements and can lead to improved asthma outcomes. If we use number of escalations as a proxy for number of emergency department visits saved in this pilot, there are huge cost savings for the hospital and value from this program. Future studies should measure actual cost savings as a result of the RPM program while incorporating the cost of the program and operationalization.

However, there are certain situations in which in-person visits are more appropriate due to urgency, a person’s underlying health conditions, or the fact that a physical examination or laboratory testing is needed for medical decision-making. Telehealth may not be ideal when addressing sensitive topics, especially if there is patient discomfort or concern for privacy. Limited access to technological devices (eg, phones, tablets, or computers) or connectivity on the part of health care providers or patients may make telehealth infeasible for some people. This may be especially true for those living in rural settings. Depending on the platform used, some health care workers or patients may be less comfortable with using the technology and may prefer an in-person visit. In some cultures, virtual visits may not be readily accepted in lieu of in-person visits by health care workers or patients [9].

The success of the RPM system also depends on the data quality. Even if the system, the sensors, and the network are running fine, the reliability of the data provided to the diagnosing practitioner would be dependent on the accuracy of data from the system. This may require verification and calibration of the RPM system from time to time in order to maintain reasonable accuracy [10].

RPM comes with a lot of promise and has evolved into a finer service than telehealth. It is going to continue to evolve with time in terms of growth and innovation.

### Conclusions

In this pilot study, we demonstrated the feasibility of implementing a novel RPM platform in patients with asthma. Our platform showed high patient engagement and satisfaction, and it provided physicians with real-time patient data and home spirometry data to evaluate patients remotely and aid in their clinical decision-making. Respiratory remote monitoring platforms such as this one have the potential to reduce thousands of emergency department visits and hence reduce the economic burden of respiratory diseases on the US health care system.

## Appendix

Multimedia Appendix 1 Baseline, tobacco, and Asthma Therapy Assessment questionnaires.

## Abbreviations

ATAQ: Asthma Therapy Attack Questionnaire
CDC: Centers for Disease Control and Prevention
RPM: remote patient monitoring

## Data Availability

The data sets generated or analyzed during this study are available from the corresponding author on reasonable request.
