# Supplementary material for: Impact of Remote Patient Monitoring Platform on Patients With Moderate to Severe Persistent Asthma: Observational Study
Source: JMIR Form Res. 2023 Dec 28;7:e51065. doi: 10.2196/51065 (PMC10784973; doi:10.2196/51065)
Supplement: Multimedia Appendix 1 [file formative_v7i1e51065_app1.pdf]

Section S1: Baseline Questionnaire

1. On a scale of 1-5 with 1 being not easy at all and 5 being very easy, how easy was entering your medication in the app?
2. On a scale of 1-5 with 1 being not easy at all and 5 being very easy, how easy was it to view or modify your Asthma Action Plan?
3. On a scale of 1-5 with 1 being not easy at all and 5 being very easy, how easy was entering your daily check-ins?
4. On a scale of 1-5 with 1 being not easy at all and 5 being very easy, how easy was it to send a message to Keva Health Support from the app?
5. On a scale of 1-5 with 1 being not easy at all and 5 being very easy, how easy was it to access and view the Learn section?
6. Did you understand your engagement report on the Home screen?
7. What features in the app did you find most useful?
8. How often do you plan to check-in?
9. How many Asthma-related ER Visits did you have in the past 3 months?
10. How many Asthma-related IN PERSON visits to the doctor's office did you have in the past 3 months?
11. How many Asthma-related TELEHEALTH visits to the doctor's office did you have in the past 3 months?
12. What % of time were you able to take your medication as prescribed by your doctor in your action plan? Please select a number from 0 to 100% (0 means none of the time, 50% means half of the time, and 100% means all the time).

Section S2: Tobacco Questionnaire

Health Care Maintenance Task: Please review your smoking status and make sure it is up to date. (Former smoker, Never smoked, Smoked everyday)

Section S3: Asthma Therapy Assessment questionnaire (ATAQ)

1. In the past 4 weeks did you miss any work, school, or normal daily activities because of your asthma? (1 point for YES)

2. In the past 4 weeks, did you wake up at night because of your asthma? (1 point for YES)

3. Do you believe your asthma was well controlled in the past 4 weeks? (1 point for NO)

4. Do you use an inhaler for quick relief from asthma symptoms? If yes, what is the highest number of puffs in 1 day you took of this inhaler? (1 point for more than 12)
